# A Review Study on Molecularly Imprinting Surface Plasmon Resonance Sensors for Food Analysis

**DOI:** 10.3390/bios14120571

**Published:** 2024-11-25

**Authors:** Bahar Bankoğlu Yola, Neslihan Özdemir, Mehmet Lütfi Yola

**Affiliations:** 1 Department of Engineering Basic Sciences, Faculty of Engineering and Natural Sciences, Gaziantep Islam Science and Technology University, Gaziantep 27000, Turkey; bahar.bankogluyola@gibtu.edu.tr; 2 Department of Machinery and Metal Technologies, Merzifon Vocational School, Amasya University, Amasya 05300, Turkey; neslihan.ozdemir@amasya.edu.tr; 3 Department of Nutrition and Dietetics, Faculty of Health Sciences, Hasan Kalyoncu University, Gaziantep 27000, Turkey

**Keywords:** surface plasmon resonance, molecularly imprinted polymer, sensor, food application

## Abstract

Surface plasmon resonance (SPR) sensors have emerged as a powerful tool in biosensing applications due to their ability to provide sensitive and real-time detection of chemical and biological analytes. This review focuses on the development and application of molecularly imprinted polymer (MIP)-based SPR sensors for food analysis. By combining the high selectivity of molecular imprinting techniques with the sensitivity of SPR, these sensors offer significant advantages in detecting food contaminants and other target molecules. The article covers the basic principles of SPR, the role of MIPs in sensor specificity, recent advancements in this sensor development, and food applications. Furthermore, the potential for these sensors to contribute to food safety and quality control was explored, showcasing their adaptability to complex food matrices. The review concluded the future directions and challenges of SPR-MIP sensors in food analysis, emphasizing their promise in achieving high-throughput, cost-effective, and portable sensing solutions.

## 1. Introduction

Biosensors are one of the most preferred methods in many fields, especially in the food and medical industries, due to their ability to provide more sensitive and stable results than traditional methods toward very small amounts of analytes. Consequently, some numerous studies have been conducted in the literature on biosensors with new applications. SPR biosensors, which have been widely used in recent years, are among the most common applications due to their many known advantages. The greatest advantages of these biosensors are that they work at the nanoscale and can be easily applied at visible optical frequencies [1,2,3,4]. 

**Figure 1:**
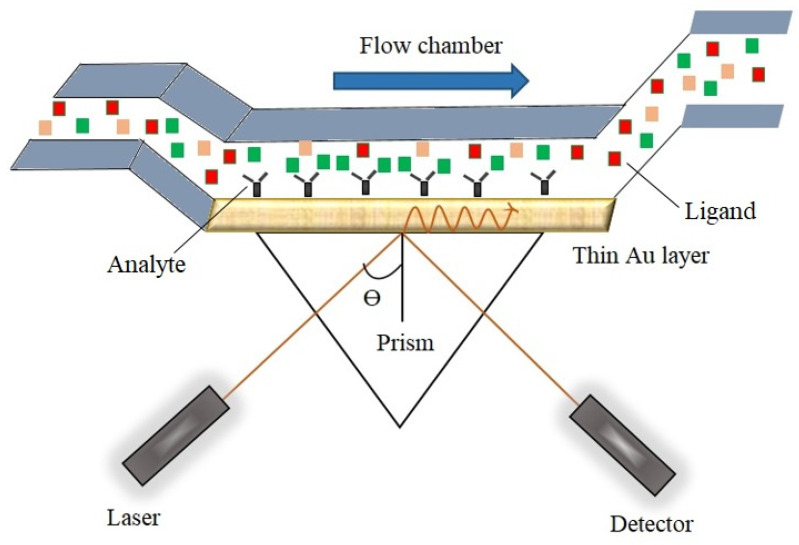
Operating mechanism of the SPR sensor.

The working principle of SPR (Figure 1) is based on the polarized light sending to a sensor surface covered with a thin metal film (mostly gold or silver) and observing the change in the reflection intensity of the light. Light striking the sensor surface at a certain angle excites plasmons as a result of reflection by the metal, leading to the SPR event. By adjusting the angle of the polarized light, a significant decrease in the intensity of the reflected light is observed. This decrease in light intensity is explained by the interaction of p-polarized light photons with the free electrons in the metal film, causing the electrons to create wave-like vibrations (resonance). The intensity of the reflected light is monitored, and the angle at which the highest decrease in intensity is observed is defined as the resonance angle or SPR angle [3,5,6,7].

Surface plasmons (SPs) were first discovered by a scientist named R. W. Wood at Johns Hopkins University in Baltimore, USA, between 1902 and 1912. During his studies, Wood noticed dark and light bands in the light spectrum that he could not explain. In 1941, Fano concluded that these bands were related to surface (lattice) waves. In 1958, Thurbadar observed a sharp decrease in reflection while conducting studies on the illumination of thin metallic films on a substrate. However, he did not attribute this decrease to SPs. Finally, in 1968, Otto explained that the decrease in reflection observed when using the Attenuated Total Reflection (ATR) method was due to SPs [6,8].

In the same year, two scientists named Kretschmann and Raether determined that SPs could be excited using a different configuration of the ATR method. As a result of the significant contributions of these three scientists and their studies, SPs were introduced to the modern field of optics. By the late 1970s, SPs were finally applied to biological and chemical mechanisms. Pharmacia, which began SPR applications in 1980, established a company known as Pharmacia Biosensor AB, after approximately four years, to produce and market devices in this field. When examining the literature of today, it is clear that this field, which began with these early studies, has made significant progress and evolved into a multidisciplinary field, now integrated into the pharmaceutical industry, food industry, and medical world [7,8].

The SPR biosensor consists of two basic elements. The first is an optical system that facilitates the excitation of plasmons on the surface. The second is biomolecular recognition elements that identify the target analyte. Surface plasmons (SPs) detect the binding of the analyte by sensing changes in the dielectric properties (i.e., refractive index). The choice of metal used is crucial for the success of the method. Ferreira de Macedo and his colleagues used gold nanoparticles to obtain a more intense signal. Commonly preferred metals include gold, silver, copper, aluminum, sodium, and indium, with gold being the most preferred. This is because gold exhibits a distinct and easily measurable resonance signal in the near-IR spectrum. Additionally, gold is resistant to oxidation and contamination. In contrast, indium is expensive, sodium is too reactive, and silver is prone to oxidation, all factors that further favor the use of gold [4,9,10,11,12,13,14].

In recent years, SPR technology has made significant progress thanks to various studies conducted worldwide. At this stage, SPR devices capable of detecting specific analytes, as well as multi-parameter SPR devices, have been developed. Additionally, research has begun on the production of portable devices. Due to the effective results of SPR technology in various scientific fields, there is great interest in developing multi-channel, multi-parameter, high-throughput, and high-integration-density devices that allow for simultaneous measurements [4,12,14].

Molecular imprinting is an innovative method that produces the desired chemical species with high selectivity and sensitivity through three stages: (i) precomplex formation, (ii) polymerization, and (iii) the removal of the target molecule. In the first stage, a complex is formed between the template molecule and the monomer. A crosslinker, which provides rigidity to the MIP structure, is also added in this stage. In the second stage, the template–monomer complex formed in the precomplex stage undergoes polymerization through chemical bonds under the conditions relating to the template molecule, the monomer, and the initiator molecule. In the third and final stage, a completely new three-dimensional MIP structure, which carries the characteristic features of the template molecule, is created [10,15,16,17,18]. Moreover, MIP is regarded as a promising method for creating next-generation synthetic polymeric receptors due to its porous structure, which provides specific recognition capabilities and numerous advantages. Several benefits contribute to the widespread use of MIPs, including low cost, high stability, easy synthesis, the ability to remain active for over a year when the proper storage conditions are maintained, and reusability for various studies [10,11,12,15,16,17,18,19]. In the structure of an MIP, the template recognition site must be properly positioned. Especially, due to the high surface-to-volume ratio belonging to MIP structures, including the nanoparticle size, the binding energy and ability to interact with the template molecule are increased, while also providing easier access to the recognition site [15].

The first known information about molecular imprinting applications dates back to 1931. During Polyakov’s studies, the first application of silica gel that exhibited a new absorption property was referred to as “molecular imprinting”. In the following years, Pauling proposed the principle of complementarity between excess antigens and antibodies in the imprinting process. This journey gained new dimensions in 1972 with the design of a new MIP based on covalent imprinting for D-glyceric acid by Wulff and his colleagues. In 1978, Shea discovered template synthesis of macromolecules and the selective functionalization of an organic polymer. In 1981, Mosbach announced new MIP structures, including non-covalent imprinting. This method, which has made significant progress over time, holds substantial value in today’s scientific world [20,21].

This work is a collective and comprehensive review that describes the definition and principles of molecularly imprinted SPR sensors, including current techniques and developments, as well as illustrating MIPs and SPR applications for food applications.

## 2. Principle of Surface Plasmons (SPs)

**Figure 2:**
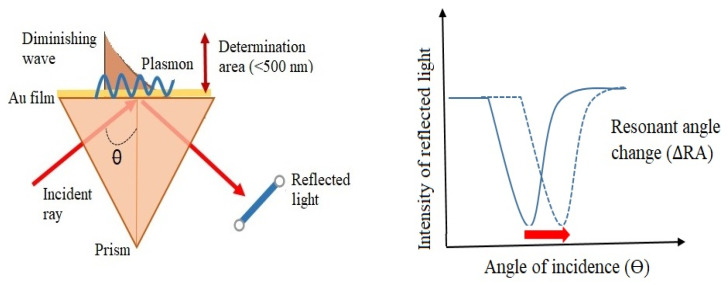
Formation of surface plasmons.

The principle of surface plasmon (SP) formation is based on p-polarized light sent to a metal surface containing plenty of free electrons in its structure, which excites these free electrons. As shown in Figure 2, in a system consisting of two interfaces, the first surface is a metal rich in free electrons, while the other is a material with dielectric properties. Surface plasmons, also known as electron density waves, propagate between these two surfaces and exhibit harmonic oscillations. In this system, the metal enables the production and accumulation of SPs on a prism, which is also in contact with the dielectric surface. The role of p-polarized light in the system is to interact with photons, propagating as waves and generating the observed electromagnetic waves [4,6,9,11].

The situation in which the light used in the formation of surface plasmons (SPs) is assumed to be completely reflected is called total internal reflection. However, not all of the light is reflected; the portion that is not reflected interacts with the SPs, resulting in the formation of a low-refractive-index area known as the evanescent field. When the photons in the SPs and the evanescent field reach equilibrium, surface plasmon resonance occurs. The angle at which this event takes place is referred to as the resonance angle or SPR angle [4,22].

Thanks to the device, known as a sensorgram, all changes occurring on the sensor surface can be monitored. These changes can manifest as a variation in the incident angle or a change in wavelength. Observing a decrease in the graphs obtained from the sensorgram indicates the formation of surface plasmon resonance. The device measures the SPR angle in resonance units (RU). Several factors affect the SPR angle, including the properties of the metal used, the wavelength of the p-polarized light, and the refractive index of the dielectric material. The location and shape of the curve resulting from the formation of SPR are influenced by the metal used; therefore, metal selection is a crucial step. Metals such as silver, gold, aluminum, and copper can be utilized to produce surface plasmons. However, each metal has its own advantages and disadvantages, making gold the more frequently preferred option. In fact, when SPR curves are examined, silver exhibits a sharper decrease compared to other metals, while gold produces a broader curve, and aluminum shows the widest curve. Although silver offers better sensitivity than gold, it is chemically unstable [4,23].

Another factor affecting the SPR curve is the thickness of the metal surface. If the metal surface is thicker than the optimal value, the curve can become shallower and may eventually fade. Conversely, if the metal surface is thinner, the curve can be wider. Thus, a thickness of 50 nm appears to be ideal for gold surfaces. In addition, the shear sensitivity of the dielectric refractive index on the metal–dielectric interface can provide a biosensing analysis. A shift in the resonance angle is related to the change corresponding to the dielectric constant. The sensor surface is sensible to the biomolecular interactions between proteins or nucleic acids when the binding takes place. The refractive index of the dielectric can deviate owing to mass changes and the binding of biomolecules on the sensor surface. Thus, the binding rate decreases over time, until it reaches a point where the rates of combination and separation events are equal. Another factor affecting the refractive index is the temperature of the surface, changing the SPR sensitivity. This sensitivity variation can be used for the determination of the binding kinetics of biomolecules and affinity degree [4,13,23,24].

## 3. SPR System

SPR devices consist of three basic elements: an optical system, a liquid system, and a sensor chip. These three components, along with the degree of automation, play an active role in differentiating the devices. In the system, the sensor chip is positioned between the liquid system and the optical system, creating a barrier between them and allowing interactions to occur directly on the liquid system side. Different methods are used in SPR systems to determine the shift rates of the SPR angle (resonance angle), with prisms, gratings, and optical waveguides being the most important. Among these, prisms configured in the Kretschmann setup are more commonly preferred in device production. In this prism configuration, p-polarized light is directed at a thin gold sensor surface and reflected to a detector that measures intensity using a camera or photodiode. In systems utilizing the grating coupler configuration, the surface to which the light is directed has a lower refractive index. Although each configuration operates differently, they all aim to directly and simultaneously measure the refractive index changes occurring on the sensor surface [9,11,12,13,14,25].

SPR sensors are capable of measuring very low concentrations of chemical and biological compounds. When chemical or biological compounds bind to the sensor surface, they replace the electrolytes, resulting in a change in the refractive index. It is known that the refractive index of water molecules is lower than that of protein molecules. At this point, it is important to emphasize that SPR devices offer high sensitivity in their measurements [26].

## 4. Molecularly Imprinted Polymers

**Figure 3:**
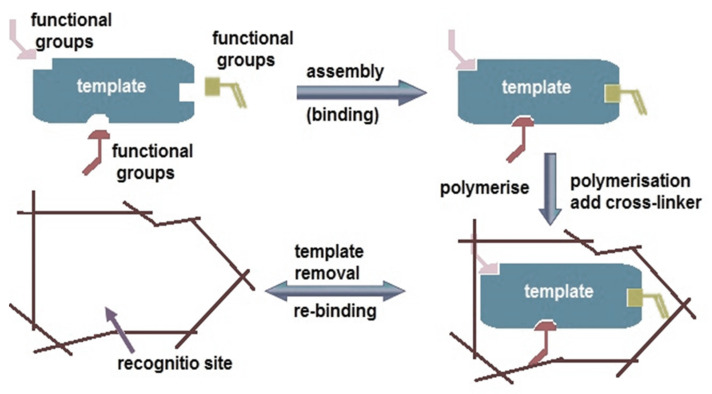
Preparation process of molecularly imprinted polymers.

The molecular imprinting technique is an important method that has been used in sensor applications in recent years. It produces a polymeric network toward the template molecule. MIPs are also synthetic materials formed by the polymerization of monomer compounds in the presence of a template molecule using this technique. The formation of MIPs occurs in three stages, as shown in Figure 3. The first stage begins with the interaction between the monomer and the template molecule. In the second stage, the crosslinker and the initiator react to form the MIP. In the third and final stage, the template is separated from the polymer, resulting in cavities that exhibit high selectivity for the target molecule. During the design phase of the study, selecting a monomer that is compatible with the physical and chemical properties of the target molecule significantly impacts the study’s selectivity. The interactions between the polymer cavities and the target molecule directly affect this selectivity [10,11,12,13,14,16,17,18,19,27,28,29,30].

When examining the interactions between the template molecule and the monomer, three main methods stand out: (i) covalent imprinting, (ii) semi-covalent imprinting, and (iii) non-covalent imprinting. The non-covalent imprinting method is generally preferred over the covalent imprinting method. Although the covalent imprinting seems advantageous in terms of creating more binding sites, the difficulty of breaking covalent bonds and the low binding affinity associated with this method make it less favorable. In the non-covalent imprinting method, the weak electrostatic interactions occur between the monomer and the template molecule. While this method may have fewer binding sites, it allows for easier removal of the template molecule. Additionally, it is generally easier to find functional monomers for the non-covalent imprinting method in comparison with the covalent imprinting method. The semi-covalent MIP method was first applied by Whitcombe, Rodriguez, Villar, and Vulfson in 1995 as a combination of covalent and non-covalent methods. This approach can incorporate the specific recognition properties of the covalent and the non-covalent methods. Thus, the semi-covalent method has been widely applied in recognition studies due to its convenient working conditions [13,14,17,18,21,30,31,32,33,34].

**Figure 4:**
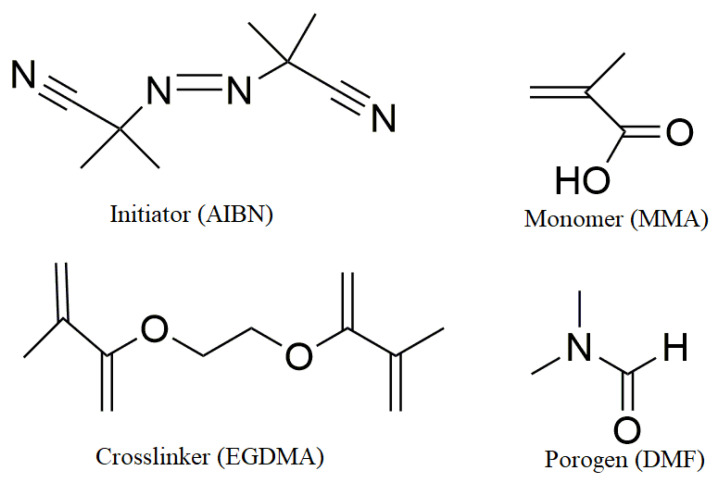
Structures frequently used in the synthesis of MIPs.

The template molecule (analyte), functional monomer, crosslinker, solvent, and initiator used in MIP synthesis are important parameters that contribute significantly to the success of the system. The monomer, initiator, porogen, and crosslinker frequently used in MIP synthesis are given in Figure 4 [16,32,34]. The analyte participates in the polymerization process alongside the monomer molecule. This stage is one of the critical points in the system. The analyte used must be non-reactive and stable under the reaction conditions, such as temperature and UV exposure. The used analyte molecules with some functional group for determination in real samples can include amino acids, carbohydrates, proteins, nucleotide bases, hormones, heavy metals, drugs, and pesticides [16,32].

The most important factor in the success of the molecular imprinting method is the monomer selection. Especially, we expect the selected monomer to interact specifically with the analyte molecule to be analyzed and form a complex. Two known methods are employed for this purpose. The first method involves using a computer application to determine the most compatible mole ratios of the template and functional monomer. The second method entails identifying suitable monomers through a semi-empirical approach, and methacrylic acid is frequently used as a monomer in this context [2,16,17,32].

Crosslinking is important for the stable formation of the polymer matrix in imprinted polymers and used to control the morphology of the polymer matrix. In addition, it stabilizes the binding sites specific to the imprinted template molecule and ensures that the polymer matrix retains its molecular recognition properties. When selecting a crosslinker, the mole ratio between the crosslinker and the monomer is crucial. This mole ratio plays an important role in the binding regions. Deviating from the ideal ratio—whether too low or too high—can negatively impact the binding regions and hinder the success of the system. The most commonly used crosslinkers are ethylene glycol dimethacrylate (EDMA) and divinyl benzene (DVB) [16,32,35].

The solvent ensures that the chemicals used in polymerization are in a single phase and homogeneous, and it is responsible for pore formation in macroporous polymers during the polymerization phase. In addition to these benefits, the solvent helps to prevent the formation of by-products by controlling heat conduction, thereby contributing to the fast mass transfer into the polymer during the rebinding [16,32].

Radical polymerization can be initiated by thermal decomposition of radical initiators. In cases where the non-covalent interactions between the monomer and the template molecule are very weak, very high temperatures are not reached. Photochemical initiators such as 2,2′-isobutyronitrile (AIBN), which are effective at low temperatures, are preferred [16,30,32].

When the polymerization stage is reached, the polymerization is achieved by selecting the appropriate method in the presence of the analyte, monomer, crosslinker, and initiator. The polymerization is categorized based on the used mechanisms, including the analyte–monomer type and crosslinker. These categories can be presented as bulk, precipitation, emulsion, and suspension polymerization. Then, the structural shape and size of MIPs are determined according to the preferred imprinting type. MIPs can be structurally obtained in the form of films or nanoparticles. Recent studies indicate that MIPs in nanoscale sizes have been developed to enhance sensitivity. The bulk method in polymerization is one of the simplest imprinting techniques; however, it is known that the polymers consist of microspheres with larger and unstable particle sizes. One of the most ideal polymerization methods for synthesizing MIPs in nanoparticle form is the precipitation process, which yields nanoparticles with more stable particle sizes and high efficiency. During this process, the MIP undergoes a slow precipitation in the presence of a mixture of crosslinker, monomer, and analyte, in accordance with the principles of the synthesis method. In the emulsion polymerization process, a mixture of crosslinker, monomer, and analyte is again present. However, unlike the precipitation process, this mixture is subjected to emulsification in a liquid medium containing a surfactant, resulting in the synthesis of stable, homogeneous droplet-sized MIPs. The droplet sizes obtained can range from 50 to 1000 nm, with sodium dodecyl sulfate (SDS) being the surfactant most frequently used. A comparison of the different polymerization methods employed in the synthesis of MIPs is presented in Table 1 [16,30,32,34].

The final stage of MIP synthesis is the removal of the template molecule. This stage is critically important for the success of the system. Current methods for the removal of the template molecule are detailed in Table 2. Among the parameters affecting the template molecule removal, the minimal processing time and the preservation of the polymer’s characteristic properties can be identified. Additionally, it is advisable to avoid complex applications to prevent negative outcomes, such as swelling, shrinkage, or damage to the pore spaces formed during the removal process [16,33].

There are many direct and indirect methods known to verify that the template removal process has been carried out correctly. Direct testing methods, such as checking for template residue in the extraction solvent, assessing the physical and chemical properties of the MIP, or using electroanalytical methods, can be employed. Checking for template residue in the extraction solvent is conducted using established techniques, such as UV-Vis, fluorescence, or NMR spectroscopy. Direct control methods are preferred due to their ease of application and accessibility. However, if these methods are not effective, indirect testing methods, such as measuring the electrochemical current of a redox probe in solution, analyzing the fingerprints of elements, employing X-ray photoelectron spectroscopy (XPS), or examining functional groups via IR spectra, can be utilized [16,33].

The science of imprinted nanomaterials, which are formed through the combination of nanoscience and imprinting technology, can minimize the negative aspects of known methods and provide several advantages, such as higher binding affinity, easy removal of the template molecule, more binding sites, and more efficient imprinting processes. It has also been reported that MIP nanoparticles exhibit lower mass transfer resistance because they have a higher surface-to-volume ratio compared to MIP thin films [32].

## 5. Introduction to MIP-Based SPR Sensors

Molecularly imprinting polymers (MIPs) are prepared to selectively recognize analyte molecules by forming a specific structure through the “molecular imprinting” process. In this process, the polymeric network is shaped around the analyte molecules, enabling it to bind only to a specific analyte. The SPR method can monitor these binding interactions by detecting shifts in optical signals. When MIPs bind to analyte molecules, these interactions induce measurable changes in the plasmon resonance, allowing for the real-time detection of molecular interactions. However, there are some limitations in the application of MIP-based SPR sensors. Especially, the small size of the imprinted molecules makes it difficult to successfully measure refractive index changes, leading to a decrease in the process’s capacity [9,14,16,17,25,30,34]. Despite these known challenges, many studies have been presented in the literature. The first known study on MIP-based SPR biosensors was conducted by Lai et al. in 1998, focusing on the determination of theophylline, caffeine, and xanthine in an aqueous solution. In the experimental phase, MIPs that underwent preliminary preparation processes (milling and sieving) were attached to a silver film and incubated in the sample solution for one hour. After drying the films, the shifts in the SPR angle were measured based on analyte concentration. The linear dynamic range reached levels up to 6.0 mg/mL, with the detection limit for theophylline determined to be 0.40 mg/mL. The sensor exhibited a shelf life of 3-to-5 years. For selectivity studies, eight different compounds physically and chemically similar to theophylline, caffeine, and xanthine were tested, resulting in high-selectivity data [40]. In addition, Table 3 shows some analytical characteristics of the earlier MIP-based SPR sensors between 1998 and 2007.

### 5.1. Current Food Applications of MIP-Based SPR Sensors Between 2016 and 2024

In a study conducted to detect the thiram pesticide, which has been used for many years to control fungal diseases in various agricultural products and dairy products, an SPR sensor based on sulfur-doped titanium dioxide nanoparticles and an MIP was designed. In the first stage of the synthesis, sulfur-doped titanium dioxide (S-TiO_2_) nanoparticles was polymerized using the sol–gel hydrolysis technique, which is a practical application. The preferred crosslinker in the study was EGDMA, the initiator was N,N′-azobisisobutyronitrile (AIBN), and the monomer was methacryloylamidoglutamic acid (MAGA). In the continuation of the study, the synthesis of the S-TiO_2_-based thiram-imprinted SPR chip was completed using UV polymerization. The synthesized SPR sensor showed linearity in the range of 1.0 × 10^−9^–1.0 × 10^−7^ M, with a limit of detection (LOD) of 3.3 × 10^−10^ M in milk samples [14].

In another study, an MIP-based SPR biosensor was developed for the detection of citrinin (CIT), a mutagenic and carcinogenic substance in rice samples. Firstly, the gold surface of the SPR chip was modified with allyl mercaptan, and CIT-imprinted poly(2-hydroxyethyl methacrylate-methacryloyamidoglutamic acid) film was formed on the SPR chip with allyl mercaptan. The developed SPR sensor was found to have a linearity of 0.005–1.0 ng/mL and an LOD value of 0.0017 ng/mL. In addition, it was reported to show high selectivity and reproducibility in the presence of other interfering agents [50].

In a study on the detection of 6-benzylaminopurine as the first artificial exogenous plant hormone in vegetables, including mung bean sprouts, soybean sprouts, cucumbers, and tomatoes, a sensor was designed and validated by combining an SPR sensor with magnetic molecularly imprinted polymer nanoparticles (MMIP NPs). Methacrylic acid and sodium p-styrene sulfonate were used as functional monomers for the synthesis of MMIP NPs. For this aim, a pre-polymerization solution was first prepared in the presence of 6-benzylaminopurine, methacrylic acid, and sodium p-styrene sulfonate. Then, the prepared Fe_3_O_4_@COOH NPs, polyvinylpyrrolidone, AIBN, and EGDMA were added to the abovementioned pre-polymerization solution and sonicated for 30 min. After polymerization reaction for 12 h, the synthesized MMIP NPs were separated by using a magnet. After the modification of the SPR surface via 3-mercaptopropionic acid, MMIPs NPs were mixed with different 6-benzylaminopurine concentrations, and 6-benzylaminopurine-captured MMIPs NPs were separated by using magnet. Finally, 6-benzylaminopurine-captured MMIPs NPs were incubated on the SPR chip surface for analytical applications. According to the results of the analyses, it was determined that the signals from the SPR sensor showed higher signal levels than those of the structural and non-structural analogs of 6-benzylaminopurine. This demonstrates that the SPR sensor exhibits high selectivity in the detection of 6-benzylaminopurine. The recovery for the four vegetables was reported to be between 93.8% and 108.6%, and the sensor showed a low LOD (3.02 pg/mL) and limit of quantitation (LOQ) (10.08 pg/mL) [51].

In a study conducted by Karslıoğlu et al., a sensor design was developed for the detection of acrylamide (AA), the most undesirable substance in starch products due to its toxic and carcinogenic properties in homemade French fries, using an MIP-based SPR sensor. In this context, after the monolayer formation of allyl mercaptane on the SPR chip, an AA-imprinted SPR chip modified with allyl mercaptane was prepared via UV polymerization, using EGDMA as a crosslinker, AIBN as an initiator, and MAGA as a monomer. As a result, it was stated that the designed MIP-based SPR sensor had an LOD value of 3.0 × 10^−10^ M and showed linearity in the range of 1.0 × 10^−9^–5.0 × 10^−8^ M. Additionally, based on the obtained results, the SPR sensor exhibits high selectivity and repeatability in AA detection in homemade French fries [52].

In a study conducted for the detection of zearalenone (ZEA), one of the toxic pollutants in cereal products, a, MIP-based SPR sensor based on a sulfur-doped g-C_3_N_4_/Bi_2_S_3_ nanocomposite was developed for rice grains. In this study, high-purity S-g-C_3_N_4_/Bi_2_S_3_ nanocomposite was synthesized using an environmentally friendly calcination method. After the modification of SPR chip surface with sulfur-doped g-C_3_N_4_/Bi_2_S_3_ via sulfur–gold affinity, the SPR sensor based on sulfur-doped g-C_3_N_4_/Bi_2_S_3_ was designed by UV polymerization in the presence of MAGA as the functional monomer, EGDMA as the crosslinker, and AIBN as the initiator. It was reported that the SPR sensor exhibited an LOD value of 0.33 ng/L and a linearity range from 1.0 to 10.0 ng/L [10].

Another study aimed to develop an MIP-based SPR sensor for the detection of Lysosome (Lyz) as a protein in complex environments due to its bactericidal and anti-inflammatory properties. Lyz-imprinted MIPs were synthesized using dopamine as both a crosslinker and monomer. 3-mercaptopropionic acid was utilized to provide non-covalent interaction via a self-assembled monolayer formation to improve the selectivity, and protein-resistant poly(2-methyl-2-oxazoline-co-ethylene imine) was used to resist nonspecific adsorption of polydopamine. According to SPR absorption studies, the LOD and LOQ values were determined to be 0.19 μg/mL and 0.58 μg/mL, respectively. Additionally, it was reported that the developed Lyz-MIPs biosensor demonstrated high reproducibility, and the recovery values in egg white were found to be between 95.3% and 105.0% [53].

Another study aimed to detect glyphosate as an active ingredient in apple samples. In this study, after the deposition of glyphosate-imprinted polymer polypyrrole (MIPpy) onto the SPR chip’s surface, it was utilized as an electrochemical surface plasmon resonance (ESPR) sensor. ESPR optically monitors the faradaic processes via the change in refractive index that occurs with a change in redox state at the electrode surface. The difficulties and limitations encountered in the development of the MIPpy-based glyphosate ESPR sensor were explained within the scope of the study. According to the data obtained in this context, glyphosate affected the electrochemical deposition process of MIP on the electrode and prevented the accumulation of polypyrrole. As an alternative method, the self-assembled monolayer formation was planned in the presence of 11-(1H-Pyrrole-1-yl)undecane-1-thiol before electrochemical deposition of MIPpy and polypyrrole without glyphosate imprints (NIPpy) was applied. The dissociation constant (K_D_) and free energy of Gibbs (ΔG^0^) values of glyphosate on MIPpy and NIPpy were calculated. For MIPpy, K_D_ and ΔG^0^ were calculated as 38.18 ± 2.33 × 10^−5^ and −19.51 ± 0.15 kJ/mol, respectively [54].

In another study, an optical chemical recognition strategy was presented for the detection of furfural as toxic and carcinogenic substance in drinking-water samples. The system consisted of the modified plastic optical fibers (POFs) combined with MIPs and SPR. The MIP deposition was performed on a microstructured POF surface which was utilized to launch the light into the SPR-POF surface. In this way, the SPR sensor did not directly contact the MIP surface or the examined sample. Thus, the sensor system was shielded from the effects of MIP thickness and refractive index. The sensor platform interacted with the furfural molecule, changing the effective refractive index of the POF and SPR conditions, and the LOD value for the detection of furfural was measured in the μg/L range [55].

In another study on the detection of 2-furaldehyde as a toxic and carcinogenic substance in wine samples, a fermented food, a system was proposed by combining the plastic optical fiber SPR sensor platform with MIPs. In the study, the effect of MIP surface thickness on the sensor system was investigated by optimizing the SPR resonance wavelength according to the refractive index (R_I_) of the sample. According to the competitive experiments, the surface layer was not too thick. Furthermore, the results indicated that the proposed SPR-MIP sensor for the detection of 2-furaldehyde in fermented beverages showed a low LOD of 0.004 mg/L. Finally, it was reported that the detection of 2-furaldehyde in fermented beverages has serious implications for product flavor and human health [56].

In another study, a polydopamine (PDA)-imprinted localized SPR biosensor design was proposed using the MIP technique for the rapid, sensitive, and specific detection of enrofloxacin (ENRO) as an antibiotic in chicken meat samples. In the study, the PDA-MIP film was synthesized by polymerizing dopamine and ENRO in Tris buffer on the surface of the LSPR sensor chip. The sensor chip was then blocked with bovine serum albumin and washed with sodium dodecyl sulfate. In order to increase localized SPR signals, the conjugates with protein molecules were served as competitors and interacted with the binding sites on the PDA-MIP film. The LOD value was obtained as 61.1 ng/mL, with a range of 25–1000 ng/mL, and the recovery rates of the system were reported to be in the range of 80.7–95.4%. Additionally, it showed good reusability, along with a low relative standard deviation [57].

In the study conducted by Bereli et al., an SPR sensor was developed using MIP technique for the detection of amoxicillin (AMOX), a known bactericidal and broad-spectrum antibiotic, in commercial and local chicken eggs. Firstly, an AMOX-based poly(hydroxyethyl methacrylate–methacrylic acid) polymeric film was synthesized on the surfaces of the SPR using the UV polymerization. The polymeric film was then characterized using ellipsometry, contact angle analysis, and atomic force microscopy. According to the measurements, the ellipsometric thickness of the chip surfaces of the AMOX-imprinted SPR was measured as 35 ± 0.9 nm. The LOD for the SPR sensor was reported as 0.0005 ng/mL, with the linearity range of 0.1–10 ng/mL. Finally, validation studies of the system were carried out using the LC-MS/MS method [58].

In a study conducted to detect aflatoxin B1 as a highly toxic substance in various food samples, such as peanut and corn, an aflatoxin B1-imprinted SPR sensor was prepared. For this aim, aflatoxin B1, as a template molecule, and N-methacryloyl-L-phenylalanine, as a monomer, were precomplexed. Then, this complex solution was mixed with HEMA and gold nanoparticles. After the modification of SPR gold surface with allyl mercaptan, the dropping treatment of the complex solution that included HEMA and gold nanoparticles was performed on SPR gold surface with allyl mercaptan. Then, the polymerization process was carried out by using UV light. The proposed sensor exhibited a wide linearity range from 0.0001 to 10.0 ng/mL and low LOD value (1.04 pg/mL) [59].

In a study conducted by Rahtuvanoğlu et al., the design and characterization of a proposed MIP-based SPR biosensor for the detection of histamine in canned tuna samples were described. Histamine is one of the most common biogenic amines found in food products, particularly in protein-rich and fermented foods. In the study, histamine-imprinted nanoparticles were synthesized using mini emulsion polymerization. Subsequently, the histamine-imprinted nanoparticles were coated onto the gold surfaces of the SPR chip to modify the chip surfaces. After the coating process was completed, the kinetic analyses of the system were performed over a wide concentration range of 0.001–10 μg/mL, and the LOD value was determined to be 0.58 ng/mL. According to the obtained results, the proposed SPR chip system demonstrated high selectivity and sensitivity for the detection of histamine. Additionally, the advantages of the system, such as easy preparation, simple operation, and low cost, were noted. Histamine-detection studies were compared with the analysis results obtained using LC-MS/MS, and excellent recovery for the proposed sensor system was concluded [60].

In a study conducted by Çakır et al., a molecularly imprinted SPR sensor was developed for the determination of 2,4-dichlorophenoxyacetic acid (2,4-D), one of the most common herbicides, in apple samples. In the study, 2,4-D-imprinted polymers were synthesized in the presence of ethylene glycol dimetacrylate-N-metacryloyl-(L)-tryptophan methyl ester as the monomer, EGDMA, and an 2,4-dichlorophenoxyacetic acid analyte molecule. The non-imprinted polymers were prepared without the 2,4-dichlorophenoxyacetic acid analyte molecule. After the modification of the SPR gold surface with allyl mercaptan, the dropping treatment of the complex solutions including MIP and NIP polymers was separately performed on the SPR gold surface with allyl mercaptan. Then, the photopolymerization process was carried out by using UV light. The sensor showed a linearity range of 0.23–8.0 nM and a low LOD value (24.57 ng/L) [61].

In a study conducted for the comparative determination of pesticides in drinking-water samples, SPR sensor chip nanofilms were prepared using the molecular imprinting technique. The affinity and kinetic binding properties of pesticides were then investigated by assessing the binding ability of pesticide-imprinted and unimprinted sensor chips using the SPR sensor. The selectivity of the pesticide-imprinted nanofilms was evaluated against the SPR sensors through comparative adsorption experiments. As a result of the experiments, it was noted that pesticide-imprinted nanofilms exhibited higher sensitivity and selectivity in comparison with unimprinted sensors. Accuracy and sensitivity assessments of the developed SPR sensor for the determination of dimethoate and carbofuran were conducted by comparing data from three different concentrations (50, 250, and 1000 ng L^−1^) and six repeat experiments with the same environmental water matrix. The LOD for dimethoate and carbofuran was determined as 16.92 ng L^−1^ and 20.47 ng L^−1^, respectively. In addition, the SPR sensor systems demonstrated good recovery values, ranging from 90.0% to 95.0%, for both pesticides [12].

Another study focused on the design of a nanoMIP–SPR sensor system, which combines MIP-based nanostructures with SPR sensor technology for the sensitive and rapid detection of vancomycin, a glycopeptide antibiotic, in milk samples. The developed nanoMIP–SPR sensor enabled the quantification of vancomycin with an LOD of 4.10 ng mL^−1^. The recovery value was also reported to be between 85.0% and 110.0%, with relative standard deviation (RSD) values of less than 7.0%. The affinity of the developed nanoMIP–SPR sensor for the analyte was found to be superior compared to other synthetic and natural receptors, with a dissociation constant of 1.80 × 10^−9^ M. Furthermore, the study highlighted that the developed sensor system streamlines the experimental processes for detecting milk contaminants, making it simpler and faster than other known methods, while also demonstrating high selectivity and sensitivity [62].

In a study aimed at detecting α-casein as the most common protein allergen, the researchers developed a nanoMIP-imprinted SPR sensor for the identification of bovine α-casein residues in milk samples. Initially, molecularly imprinted polymer nanoparticles (nanoMIPs) with high affinity toward bovine α-casein were synthesized using the solid-phase printing method. This method is an easy sample preparation technique for analyte preconcentration and sample matrix removal, and its basic advantage is the miniaturization of the extraction procedure using molecularly imprinting polymers. Then, nanoMIPs were incorporated into SPR system, and the results indicated that the nanoMIPs exhibited a high binding affinity and selectivity toward α-casein, with a dissociation constant (K_D_ = 1.0 × 10^−8^ M). The LOD value for α-casein in the sensor SPR system was also reported as 0.127 ppm. Additionally, the recovery value for the extracted wastewater samples ranged from 87.0% to 120.0%. Ultimately, the developed SPR sensor system provided a reliable and effective method for detecting food allergens [63].

The detection of multiple pesticides, such as cyanazine, simazine, and atrazine, using MIP-based SPR sensor systems was presented. In the study, molecularly imprinted nanofilms were prepared using the UV polymerization method. Surface characterization of the pesticide-imprinted SPR sensor was performed using atomic force microscopy, contact-angle measurements, and ellipsometry methods. In the continuation of the study, kinetic, isotherm, and selectivity experiments of pesticide-imprinted and unimprinted SPR sensors were conducted. Real-time measurements of the SPR sensor system offered a good detection range, which was between 0.10 and 6.64 nM. Additionally, the LOD values obtained for cyanazine, simazine, and atrazine were reported as 0.095, 0.031, and 0.091 nM, respectively. It was generally determined that the imprinted SPR sensor was quite sensitive and selective. Finally, it was reported that the developed SPR sensor could be considered an alternative method to existing pesticide-detection methods due to its reusability, fast response, and ease of use [64].

It is known that wine astringency is formed as a result of the interaction between polyphenols from grapes or other sources and salivary proteins in the mouth. Since sensory analyses of wine astringency are time-consuming, variable, and costly, the development of new detection methods is very important. In the study, the localized surface plasmon resonance/molecularly imprinted polymer (LSPR/MIP) biosensor system aimed to elucidate the relationship between wine astringency and wine. Additionally, wine astringency was evaluated at the molecular and atomic levels, and the developed sensor system was compared to sensory analyses. It was reported that the developed LSPR/MIP sensor provided a linear response from 1.0 to 140.0 µM. In addition, the astringency data obtained from the sensor system were compared to those from a trained panel, and a good correlation was observed. Finally, the applicability of the developed LSPR/MIP sensor in wine production was reported [65].

Another study focused on the development and characterization of an MIP-based SPR sensor system for the detection of profenofos, a widely used organophosphorus pesticide, in drinking-water samples. In this study, the detection probe was prepared by coating a 40 nm thick silver layer onto the uncoated core of a multimode fiber. The performance tests of the sensor were conducted in the concentration range from 1.0 × 10^−4^ to 1.0 × 10^−1^ µg/L. To achieve the best performance from the developed sensor, the profenofos concentration was optimized, and it was determined that the highest sensitivity value was achieved at 12.7 nm/log (µg/L) at a concentration of 1.0 × 10^−4^ µg/L, and the LOD value of the sensor was determined to be 2.5 × 10^−6^ µg/L. Selectivity experiments of the system were carried out using different pesticides, and it was reported that the proposed system was a highly selective probe for the profenofos molecule [66].

Another study on the detection of atrazine as one of the important herbicides involved the development and characterization of a fiber-optic SPR sensor. In the study, the detection probe was created by coating the optical fiber core surface with a 40 nm thick silver layer. Additionally, atrazine, which was selected as the template molecule, was imprinted onto the silver-coated surface of optic SPR surface. The probe was characterized using the spectral interrogation method. The developed sensor was tested for atrazine concentrations in the range from 1.0 × 10^−12^ M to 1.0 × 10^−7^ M, and it was reported that the LOD was calculated to be 1.92 × 10^−14^ M, and the LOQ was obtained to be 7.61 × 10^−14^ M. Consequently, the MIP-based SPR sensor for the detection of atrazine exhibited high selectivity and sensitivity [67].

### 5.2. Other Recent Applications of MIP-Based SPR Sensors in Different Matrices, Except for Food Sample Applications, Between 2019 and 2024

MIP-based SPR sensors have exhibited superior versatility across various fields owing to their high sensitivity, selectivity, and adaptability. While important advancements have been achieved in food-related applications, the recent developments have extended their utility into diverse matrices, encompassing medical and biological samples. These innovations leverage the unique advantages of MIP-based SPR systems to address challenges such as real-time detection, low detection limits, and interference in complex sample matrices. This section highlights the recent applications of MIP-based SPR sensors in different matrices beyond food samples, focusing on their analytical performance and the implications for advancing sensor technology in broader contexts.

For example, an ion-imprinted polymer-integrated SPR sensor was prepared for the determination of copper (Cu(II)) ions in plasma and urine samples. In this study, firstly, SPR gold-surface modification with allyl mercaptan was performed via sulfur–gold affinity. After the preparation of N-methacryloyl-L-cysteine methyl ester–Cu(II) monomer complex, the precomplex was mixed with HEMA-EGDMA-AIBN solution. The dropping treatment of the complex solution was performed on an SPR gold surface with allyl mercaptan, and the bulk polymerization was completed under ultraviolet light. The sensor showed a linearity range of 0.04–5.0 µM and a low LOD value (0.027 µM) [68]. In this context, the other recent studies conducted on the other matrix applications of MIP-based SPR sensors are presented in Table 4 [69,70,71,72,73].

## 6. Conclusions

This review has demonstrated that combining molecularly imprinted polymers with surface plasmon resonance technology offers a highly effective approach to detecting contaminants in food, such as pesticides, allergens, and antibiotics. Up to now, some traditional analytical methods, such as HPLC, LC-MS/MS, and ELISA, have been frequently mentioned as the primary methods in current food applications. However, these analytical methods have some disadvantages, such as high costs, significant time requirements, too much chemical consumption, and extensive preparation processes. Since these disadvantages belonging to traditional methods are minimized in MIP-based SPR techniques, MIP-based SPR sensors outperform many traditional methods, enabling the precise detection of even trace amounts of harmful substances. Thus, the findings highlight the adaptability of these sensors in handling complex food matrices, making them a valuable tool for food safety and quality control. Another one of the most significant advantages of this MIP-based SPR method is its ability to achieve rapid, real-time detection with high accuracy.

Looking to the future, there is great potential for further improving these sensors. Optimizing the polymerization processes, enhancing the reusability of the sensors, and expanding the range of detectable substances could lead to even more cost-effective and scalable solutions. These improvements can be achieved by obtaining quantum-sized particles with high efficiency and then integrating them into the MIP-based SPR system. Moreover, the efficiency of the system can be increased by developing multi-channel detection systems, providing simultaneous analysis. Finally, MIP-based SPR sensors have the potential to become a key technology in the routine monitoring of food safety, offering a practical, efficient, and reliable method for ensuring product quality on a broad scale.

## Figures and Tables

**Table 1:** Different polymerization methods used in the synthesis of MIPs and their comparisons [2,32].

Pre-Preparation Method	Polymerization Method	Application Method	Advantages	Disadvantages
Free radical polymerization	Bulk polymerization	Polymerization of an organic solvent is achieved. It is then subjected to sieving and grinding processes.	It is a fairly simple technique.	The technical processes used in the method (sieving and grinding) require intensive effort and time. It is limited in terms of selectivity and repeatability.
	Precipitation polymerization	Polymerization occurs in a solution. When the polymer is formed, precipitation occurs. In this way, the dissolution of the polymer is prevented.	It allows the production of MIP between 50 and 100 nm.	The particle size of the MIP sizes formed is affected by environmental factors (mixing speed, temperature, etc.).
	Suspension polymerization	Polymerization occurs in an aqueous environment.	They contribute widely to the production of monodisperse MIPs. Polymerization occurs in a single step, spherical particles are formed.	The presence of surface reactive substances may pose a risk of toxicity. Particle sizes are in the hundreds-of-micrometers level, and the recognition capacity is reduced.
	Emulsion polymerization	Polymerization occurs in the presence of surface-active compounds capable of emulsifying organic and aqueous phases.	Large-scale MIP production. High yield, water solubility.	Large particle size at the microlevel. Limited printing performance and surface contamination.
	Multistep swelling	The phase of the initiator, consisting of an oil–water emulsion, plays an active role in the swelling	Spherical stable MIP production.	The process is long and requires detailed optimization at each stage.
	Electropolymerization	The synthesized MIPs are collected on the surface of the electrode material used.	It is a simple, fast, and low-cost method.	Although the process is convenient, the characterization of the method is costly. The optimization phase is complex and takes a long time.
Sol–gel polymerization	Sol–gel polymerization	This method is based on the dissolution of metal oxides in a solvent with a lower molecular weight, through hydrolysis and poly-condensation stages.	It is a method that can be applied in the current environmental conditions.	An ideal polymerization method is not known, and monomer selection is difficult.
Surface imprinting technique	Surface-imprinting technique	During the polymerization phase, thin layers of MIPs are added to the surfaces.	It provides easier access to the target molecule-binding areas.	The process is time-consuming.
Controlled living polymerization	Atom transfer radical polymerization	It is known as a new version of the Kharasch addition method, which occurs in a 1:1 ratio between alkyl halides and alkenes.	A large number of compounds are known for initiator selection. They have a structure compatible with different monomers. Reaction conditions are easy.	Toxicity can be observed in halide initiators. The reduced metal catalyst has a structure sensitive to oxygen.
	Reversible splicing–fragmentation chain transfer	A specific transfer agent is used that allows for the rapid degenerative transfer of free radicals emitted during the process.	It can be applied in some free radical polymerizations. The efficiency of the system is not determined by the performance of a catalyst or environmental factors.	The chain transfer agent has low stability in the reaction medium. In case the reaction takes place in aqueous conditions, it is simply transformed by hydrolysis or nucleophilic attack.

**Table 2:** Current removal methods used for template removal and their positive/negative effects.

Removal Method	Process Conditions	Positive Effects	Negative Effects	Reference
Solvent extraction	Aqueous or organic solvent May require temperature increase and convection adjustments Supported by acid, base, or enzymes	Mild Simple	Very time-consuming Waste of solvent	[36]
Supercritical fluid extraction	Supercritical CO_2_	Ability to penetrate deeper and faster in solid materials	Not ideal for polar components High pressure and temperature	[37]
Soxhlet extraction	Thermal treatment of MIP with extraction solvent in a Soxhlet apparatus	Simple Hybrid system	Specific apparatus Not ideal when using non-polar solvents	[38]
Microwave-assisted extraction	Solvent extraction should be performed in a microwave oven	Fast Simple	Applicable to only some polymer types	[39]

**Table 3:** Analytical characteristics of the earlier MIP-based SPR sensors between 1998 and 2007.

Analyte Molecule	Monomer	Crosslinker	Dynamic Range	Detection Limit	Reference
Theophylline	Methacrylic acid	EGDMA/chloroform	1.0–6.0 mg/mL	0.40 mg/mL	[40]
Caffeine					
Xanthine					
Theophylline	N-(N-propyl) acrylamide-methacrylic acid	N,N′-methylene bisacrylamide/acetonitrile	-	1.0 × 10^−6^ M	[41]
Sialic acid	p-vinylbenzene boronic acid-N,N,N-trimethylaminoethyl methacrylate—HEMA	EGDMA/DMF	0.1–0.5 mM	-	[42]
NAD(P)H NADP^+^	Acrylamide-acrylamidophenylboronic acid	N,N′-methylenebisacrylamide	1.0 × 10^−6^–1.0 × 10^−3^ M	1.0 × 10^−7^ M	[43]
Domoic acid	2-(diethylamino)ethyl methacrylate	EGDMA/water	5.0–100.0 μg/L	5.0 μg/L	[44]
Dopamine	Acrylic acid-N-isopropylacrylamide	N,N′-methylene bisacrylamide/DMSO	1.0 × 10^−9^–1.0 × 10^−3^ M	-	[45]
DPDPE δ-opioid receptor	n-vinyl pyrrolidone, 3-(acryloxypropyl) trimethoxysilane	EGDMA/n-butanol	5.0–350.0 pM	-	[46]
N,N′-didansyl-L-cystine Didansyl-L-lysine	2-vinyl pyridine	EGDMA/acetonitrile	0.1–1.0 mg/mL 0.01–0.30 mg/mL	-	[47,48]
Ochratoxin A	Pyrrole	Ethanol/water	0.05–0.50 mg/L	-	[49]

EGDMA, ethylene glycol dimethacrylate; DMF, dimethylformamide; DMSO, dimethyl sulfoxide; HEMA, hydroxyethyl methacrylate; NADP^+^, β-nicotinamide adenine dinucleotide phosphate; DPDPE, δ-opioid G-protein-coupled receptor antagonist.

**Table 4:** Other recent applications of MIP-based SPR sensors in different matrices between 2019 and 2024.

Target Molecule	Material/Method	Detection Range	Detection Limit	Target Matrix	Reference
Alpha-fetoprotein	Boric acid/siloxane polymer	0–1.0 ng/mL	2.72 fg/mL	-	[69]
Modafinil	ESPR	0.1–10 μM	50.0 nM	Saliva	[72]
Cortisone	Cortisone-imprinted nanoparticles	15.0–63.0 nM	1.60 nM	Surine	[70]
Metronidazole	CdTe quantum dots/ Fe_3_O_4_NPs	5.0–60.0 μM	1.28 μM	Water	[71]
Hemoglobin	Thymine base with acrylamide	4.0–64.0 nM	3.47 nM	Blood	[73]
Equine procalcitonin	Polynorepinephrine-based MIP	25.0–1000 ng mL^−1^	15.0 ng mL^−1^	Plasma	[74]
Endotoxin	Endotoxin-imprinted nanofilms	0.5–100 ng/mL	0.023 ng/mL	Plasma	[11]
Gonadorin	A polynorepinephrine-based MIP	0.81–13.0 μM	0.10 μM	Urine	[75]
L-phenylalanine	L-phenylalanine imprinted polymeric films	5.0–400.0 μM	0.0085 μM	Plasma	[76]
Troponin I	Poly-nephrine-imprinted MIP sensor	2.50–20.0 nM	0.45 pM	Human serum	[77]
4-nitrotoluene	Au nanostructures	1.0 pM–100.0 µM	1.0 pM	-	[78]
Tetracycline	Magnetic molecularly imprinted polymers nanoparticles	5.0–100 pg.mL^−1^	1.0 pg.mL^−1^	-	[79]
Etoposide	Core–shell nanoparticles-incorporated boron nitride nanosheets	1.70 × 10^−12^–1.70 × 10^−9^ M	4.25 × 10^−13^ M	Urine	[80]
Human serum albumin	Albumin-imprinted nanoparticles	0.15–500.0 nM	0.70 pM	Urine	[81]

## Data Availability

Not applicable.
